# Characterization of the Triacylglycerol Fraction of Italian and Extra-European Hemp Seed Oil

**DOI:** 10.3390/foods10050916

**Published:** 2021-04-22

**Authors:** Carmela Tringaniello, Lina Cossignani, Francesca Blasi

**Affiliations:** 1 Department of Pharmaceutical Sciences, University of Perugia, 06126 Perugia, Italy; carmela.tringaniello@unipg.it (C.T.); francesca.blasi@unipg.it (F.B.); 2 Center for Perinatal and Reproductive Medicine, University of Perugia, Santa Maria della Misericordia University Hospital, Sant’Andrea delle Fratte, 06132 Perugia, Italy

**Keywords:** hemp seed oil, fatty acids, stereospecific analysis, geographical origin, discrimination, authenticity, traceability

## Abstract

Hemp seed oil (HSO) has received considerable attention for its health properties, especially due to unsaturated fatty acid (UFA) content. In this work, the triacylglycerol (TAG) fraction of Italian and Extra-European HSO was characterized by applying an enzymatic approach, based on the use of pancreatic lipase and *sn-*1,2-diacylglycerol kinase. This procedure allows determination of the intrapositional FA% composition of TAG. The results of the stereospecific analysis are useful for deepening knowledge on HSO nutritional aspects. The high percentage of UFA (88.3–89.9%), in particular essential FA (74.4–85.9%), of HSO samples in *sn*-2 position is important for long-term health effects, but also to enhance the use of this oil as a functional ingredient in food, cosmetic and nutraceutical fields. Furthermore, the results of total and intrapositional FA % compositions, subjected to principal component analysis, were able to differentiate HSO Italian samples from Extra-European ones. Based on the obtained results, it can be stated that the stereospecific analysis represents a potent analytical tool providing the fingerprint of TAG fraction, useful to highlight possible chemical descriptors for HSO authenticity and traceability purposes.

## 1. Introduction

Hemp (*Cannabis sativa* L.) is an annual herbaceous plant that is considered one of the oldest crops in the world, capable of rapid environmental adaptation. It is a multifunctional plant for various traditional (food, textile fiber, medicine) and innovative (biomaterials and biofuels) industrial applications [1]. Actually, there is growing interest in cultivating industrial non-drug hemp for using its seeds for oil and flour production [2].

*C. sativa* L. seeds are a rich source of protein, lipids, and dietary fiber for human and animal nutrition [3]. Nowadays, commercial products containing these seeds are considered as an interesting source of bioactive compounds to use as cosmeceuticals and nutraceuticals, even if hemp seed oil (HSO), as well as a food, was used for thousands of years in Chinese medicine [4]. According to European Union legislation, commercial production and distribution of hemp (about 70 varieties) is permitted if the content of Δ-9-tetrahydrocannabinol (THC), the principal psychoactive compound, is less than 0.2% [5]. The chemical composition of seeds can differ according to the cultivar, environmental conditions, harvesting period, processing treatment, and extraction methods [6]. Recently, Irakli et al. (2019) [3] showed that protein and oil contents of industrial hemp seeds were mainly affected by genetic factors, while total phenols, tocopherols, carotenoids, and antioxidant properties, appeared to be mainly influenced by growing year and genotype.

The most interesting feature of the HSO is the high content (about 80%) of polyunsaturated fatty acids (PUFA), especially represented by linoleic (LA; C18:2n6) and α-linolenic (ALA; C18:3n3) acids, two essential fatty acids (EFA) [7], with an optimal n6/n3 ratio (3:1), beneficial for human health. It has been reported, in fact, that a low ratio is desirable in order to reduce the cardiovascular disease risk [8]. Interestingly, HSO shows the presence of γ-linolenic (GLA; C18:3n6) and stearidonic (SDA; C18:4n3) acids, generally not found in other commodity seed oils. Moreover, it has been shown that n3 polyunsaturated fatty acids (n3 PUFA) are characterized by numerous effects in humans, playing a positive role in various human diseases (i.e., obesity, cardiovascular diseases, type 2 diabetes mellitus, and cancer) [8].

Based on the above, HSO chemical characterization is of great importance for evaluating its nutritional and health properties. In addition to this, compositional data are very useful for the complete chemical characterization of HSO, there is great interest in guaranteeing the origin and quality of Italian HSO samples. It is known that the fight against food fraud requires a comprehensive approach involving the request for complete and truthful information on food products [9]. In this field, the authentication of foods, regarding origin, production, and processing, must be guaranteed.

Several authors have applied chromatographic techniques for profiling various analytes to use as hypothetical chemical markers for authentication and tracking/tracing purposes. Generally, the obtained results highlighted that a single dataset does not give sufficient classifications, while the application of statistical models improves classification results [10]. The use of FA compositional data (total and/or intrapositional) in the food authenticity field has been also reported in numerous papers [11,12,13].

To the best of our knowledge, many findings regard HSO FA composition, but little information is reported about the lipid structure. In the present study, for the first time, the FA % compositions (total and intrapositional) of triacylglycerol (TAG) fraction of HSO of Italian and Extra-European samples were investigated by using stereospecific analysis.

## 2. Materials and Methods

### 2.1. Chemicals

ATP disodium salt hydrate (Na_2_ATP), lipase from porcine pancreas (EC 3.1.1.3), *sn*-1,2-diacylglycerol kinase from *Escherichia coli* (DAGK; EC 2.7.1.107), and γ-linolenic acid (GLA, C18:3n6; ≥99) were purchased from Sigma-Aldrich (St. Louis, MO, USA). The production of deionized water (>18 MW cm resistivity) was carried out by a Milli-Q SP Reagent Water System (Millipore, Bedford, MA, USA). FAME (fatty acid methyl esters) standard mixture, named Supelco^TM^ 37 component FAME, was obtained from Supelco (Bellefonte, PA, USA). All solvents were from Carlo Erba Reagents (Milan, Italy).

### 2.2. Hemp Seed Oil Samples

Eight commercial HSO samples, from Italy (ITA, *n* = 4) and from Extra-European Union (EEU, *n* = 4) countries, were purchased in local market and stored in the dark at 4 °C until analysis.

### 2.3. Stereospecific Analysis of TAG

The TAG fraction was separated from HSO by using TLC (thin layer chromatography) plates and petroleum ether/diethyl ether/formic acid (70:30:1, *v/v*/*v*) as developing solvent. An aliquot of TAG fraction was subjected to transesterification reaction and the obtained FAME were analyzed by high resolution gas chromatography coupled with a flame ionization detector (HRGC-FID), as reported in Section 2.4, to obtain the total FA% composition (named A_t_). Another aliquot was used for the stereospecific analysis according to the procedure reported by Cossignani et al. (2016) [14].

#### 2.3.1. Pancreatic Lipase Hydrolysis

The pancreatic lipase procedure was carried out to have the FA % intrapositional composition of TAG *sn*-2 position (named A_2_) [15]. TAG hydrolysis products were separated by TLC plates with petroleum ether/diethyl ether/formic acid (70:30:1, *v*/*v*/*v*) as developing solvent. The fraction corresponding to *sn-*2-monoacylglycerols (*sn*-2-MAG) was scraped off, extracted, and subjected to transesterification. The obtained FAME were analyzed by HRGC-FID as reported in Section 2.4.

#### 2.3.2. Preparation of Sn-1,2(2,3)-Diacylglycerol (DAG)

An aliquot of TAG was subjected to deacylation with Grignard reagent [16]. The reaction products were separated on TLC plates with hexane/diethyl ether (60:40, *v*/*v*) as developing solvent to obtain *sn*-1,2(2,3)-DAG mixture, which was isolated by TLC prior to the DAGK enzymatic procedure as reported in Section 2.3.3.

#### 2.3.3. DAGK Enzymatic Procedure

Cardiolipin solution, buffered DAGK, and Na_2_ATP were added to *sn*-1,2(2,3)-DAG and incubated at 40 °C for 90 min under constant stirring. The reaction products were separated on TLC plates using chloroform/methanol/25% ammonia (65:25:5, *v*/*v*/*v*) as developing system. The band of the *sn*-1,2-phosphatidic acids was scraped off, transesterified to obtain the constituent FAME, and analyzed by HRGC-FID, as reported in Section 2.4. The obtained data provide the FA % intrapositional composition of *sn*-1,2 positions (named A_1,2_). The FA composition of the *sn*-1- and *sn*-3-positions was obtained applying the following formulas:

A_1_ = 2A_1,2_ – A_2_

A_3_ = 3A_t_ – A_2_ – A_1_

where A_1_ is the FA % composition of TAG *sn*-1 position; A_1,2_ is the FA % composition of TAG *sn*-1 and *sn*-2 positions; A_2_ is the FA % composition of TAG *sn*-2 position; A_t_ is the FA % composition of total TAG; A_3_ is the FA % composition of TAG *sn*-3 position [14].

### 2.4. Preparation of FAME and HRGC-FID Analysis

Sodium methoxide-catalyzed transesterification was used to prepare FAME [17]. The HRGC analysis of FAME was performed following the method described by Maurelli et al. (2009) [18]. Analysis was carried out using a DANI GC1000 gas chromatograph (Norwalk, CT, USA) equipped with a split-splitless injector and an FID. The chromatographic separation was carried out using a silica capillary fused column named CP-Select CB for FAME (50 m × 0.25 mm i.d., 0.25 μm f.t.; Varian, Superchrom, Milan, Italy). The initial oven temperature was 180 °C, kept for 6 min. Then the temperature was raised at a rate of 3 °C/min to 250 °C, kept for 10 min. The injector and detector temperature was 250 °C. A Clarity integration software (DataApex Ltd., Prague, Czech Republic) was used for acquiring and processing chromatographic data. The Supelco™ 37 component FAME was used for the identification of single FA, while the peak area of each FA was used to calculate the FA percentage.

### 2.5. Statistical Analysis

The results of the analyses are expressed as the mean value and standard deviation (SD) based on three replicates. Principal component analysis (PCA) was used for the differentiation and classification of samples. Data were processed and edited with Microsoft Excel 2016 (Microsoft Office, Redmond, WA, USA) and XLSTAT (version 2021.1) software.

## 3. Results and Discussion

### 3.1. Fatty Acid Composition of HSO Samples and Nutritional Quality Index

Industrial hemp has been traditionally cultivated as a source of fibers, but the growing interest in the nutritional properties of the seeds has promoted its further development, especially for the interesting FA composition. In this paper, the FA %composition of HSO samples was determined using HRGC-FID, and the results have been reported in Table 1. Eleven main FA were detected: palmitic (PA; C16:0), stearic (SA; C18:0), arachidic (C20:0) and behenic (C22:0) acids were the saturated fatty acids (SFA); palmitoleic (C16:1n7), oleic and octadecenoic acids (C18:1n9+n7), gondoic (C20:1n9) acids were the monounsaturated fatty acids (MUFA); linoleic (LA; C18:2n6), α-linolenic (ALA; C18:3n3), γ-linoleic (GLA; C18:3n6), and stearidonic (SDA; C18:4n3) acids were the PUFA. Figure S1 (Supplementary Materials) shows the total percentages of SFA, unsaturated (UFA: sum of PUFA and MUFA), monounsaturated (MUFA), polyunsaturated (PUFA), n6PUFA, and n3PUFA.

First of all, it must be taken into consideration that the quali- and quantitative FA compositions of vegetable oils, including HSO, may vary considerably with variety, climate, and growing conditions [3,19].

It is known that HSO is a highly unsaturated vegetable oil, in fact in this work UFA mean value was 88.8% for Italian samples and 89.5% for EEU samples. The SFA value ranged from 10.5% to 11.7% for ITA samples, and from 10.1% to 10.9% for EEU samples. These data are in agreement with the results shown by Siano et al. (2019) [20]. These authors studied some Italian edible hemp products (seeds, flour cake, and oil), and reported that cold-pressed HSO showed the PUFA content around 75%, followed by MUFA (13%) and SFA (about 10%). Lower values for SFA and MUFA, and higher values for PUFA were reported by other authors; for example, Ying et al. (2018) (SFA 8.6%; MUFA 10.7%; PUFA 78.6%) for cold-pressed HSO purchased in Poland [21]; Vodolazska and Lauridsen (2020) for HSO provided by a Denmark manufacturer (SFA 9.4%; MUFA 11.3%; PUFA 78.5%) [22].

The most abundant PUFA in HSO samples was proved to be LA (on average 54.7% for ITA samples and 56.9% for EEU samples), followed by ALA (from 12.3% to 17.5% for ITA samples and from 15.8% to 19.0% for EEU samples). As also reported in other papers [2,19], this study confirms that all HSO samples contained LA and ALA as major n6PUFA and n3PUFA, respectively, but it has been also reported that HSO showed a 2:1 ratio of LA and ALA [23]. The percentage of n6PUFA is very similar between ITA (57.6%) and EEU (57.5%) samples. Marzocchi and Carboni (2020) reported n6PUFA values of 52.5–55.7% (HSO from Futura 95 cultivar) and 55.6–57.0% (HSO from Carmagnola cultivar) for Italian HSO samples [19]. The abundance of LA is also confirmed in other papers. Siano et al. (2019) [20] reported LA values of about 56% for Italian cold-pressed HSO obtained from Fedora cultivar seeds, while Pavlovic et al. (2019) [1] analyzed Italian samples and reported LA mean values of 57.69% and 57.22% for Finola and Futura 75, respectively. Also, Montserrat-de la Paz et al. (2014) [24] reported for refined Spanish HSO that the main fatty acids were LA, ALA and GLA (55.05, 16.70 and 3.40%, respectively), and oleic acid (11.90%), which together comprised 88% of the total fatty acids. As regards other PUFA, in this work, Italian HSO samples showed higher contents for GLA (2.4–3.5% vs. 0.6–0.7%) and SDA (0.6–1.0% vs. 0.2%) with respect to EEU samples.

Oleic acid (+n7 isomer) was the main MUFA for all samples (12.5–15.2% for ITA samples and 11.5–14.1% for EEU ones). This content affected strongly the total MUFA percentage (on average 15.8% for ITA and 14.7% for EEU samples) because the other MUFA (palmitoleic and gondoic acids) are low in contents (0.1–0.5%). Da Porto et al. (2012) reported for HSO extracted from Italian seeds (Felina cultivar) by supercritical carbon dioxide a MUFA value of 11.12–11.63% [25], while Petrović et al. (2015) found 10.29–14.57% for various HSO from the Croatian market [26], and Kiralan et al. (2010) 12.1–16.4% for Turkish samples [27]. In some hemp varieties, the gondoic acid is present in amounts up to 0.7%, even if most varieties typically contain lower content [19,20,27]; on the contrary, Montserrat-de la Paz et al. (2014) reported 1.44% for gondoic acid [24].

The principal SFA was PA both for ITA (5.9–7.9%) and EEU (6.4–7.3%) samples, followed by SA that was represented in similar percentage in all samples (2.5–3.0% for ITA; 2.7–3.2% for EEU). Marzocchi and Carboni (2020) reported higher SFA values for Italian samples at different harvesting times (12.1–16.7 mg/100 mg FAME for Futura 75; 11.6–12.2 mg/100 mg FAME for Carmagnola) [19]. Da Porto et al. (2012) reported an SFA value of 7.51–7.77% [25], Petrović et al. (2015) found 9.43–11.33% for Croatian samples [26], Kiralan et al. (2010) 9.4–10.6% for Turkish samples [27], and Montserrat-de la Paz et al. (2014) a value of 11% for Spanish HSO samples [24]. Anwar et al. (2006) reported values up to 8.27% for PA of HSO samples indigenous from three agroecological Pakistan regions, up to 60.50% for LA, up to 20.0% for ALA, while GLA ranged from 0.63% to 1.65% [28].

Interestingly, in this work some statistically significant differences (*p* < 0.01) based on total TAG % composition were found between the two groups of samples (ITA and EEU) for linoleic, γ-linolenic, α-linolenic, gondoic, and stearidonic acids.

Since HSO contains a high proportion of PUFA, this vegetable oil can undergo degradation due to oxidation after exposure to air, light, and/or high temperatures. For this reason, HSO preferably should be consumed cold, while should not be used for frying or baking [29]. Nutritionists suggest that daily requirements should be in the range 9–18 g for LA and 6–7 g for ALA. These contents could be obtained with the consumption of 3–5 tablespoons of HSO. The high amounts of PUFA, in particular LA and ALA, may have positive nutritional effects and physiological implications on the prevention of coronary heart diseases and cancer [8]. The average Western diet is deficient in GLA, so HSO provides this interesting FA with high pharmaceutical value for degenerative chronic diseases [29]. SDA is another noteworthy FA, being a precursor to long chain n3PUFA, important compounds for development, health, and immunity of infants. For example, recently Vodolazska and Lauridsen (2020) reported that piglets were able to convert ALA and SDA, obtained *via* the sow milk intake (5% HSO diet), to eicosapentaenoic acid (EPA; C20:5n3) and docosapentaenoic acid (DPA; C22:5n3) and hypothesized that HSO has the capability to benefit piglets during early life [22].

To better evaluate the quality of FA% composition, some ratios (UFA/SFA; MUFA/SFA; PUFA/SFA; n6/n3 PUFA) and indexes were calculated (UI, unsaturation index; AI, atherogenic index; COX, calculated oxidability value; TI, thrombogenic index; HH, hypocholesterolemic/hypercholesterolemic index) using the formulae reported by Ratusz et al. (2018) [30]; the values are shown in Table 2. It is known that some FA can help to prevent (n3PUFA, n6PUFA, and MUFA considered as antiatherogenic and antithrombogenic FA) or promote coronary thrombosis (C14:0, C16:0, and C18:0 considered as thrombogenic FA) and atherosclerosis (C12:0, C14:0, and C16:0 considered as atherogenic FA) based on their effects on LDL concentration and serum cholesterol [31].

The analyzed HSO samples were characterized by a high PUFA/SFA ratio (6.1–7.2 for ITA and 6.8–7.5 for EEU samples), which is considered favorable for the reduction of serum cholesterol and atherosclerosis, and prevention of heart diseases. Similar values have been reported by Siano et al. (2019) [20] and by Montserrat-de la Paz et al. (2014) [24], while lower values (3.87–5.65 for Futura 75; 5.70–6.08 for Carmagnola) were found by Marzocchi and Carboni (2020) [19], and higher values (7.0–8.1) were reported by Kiralan et al. (2020) [27].

The ALA and SDA were the main n3PUFA, while LA and GLA are the main n6PUFA. They directly affected the n6/n3 PUFA ratio. It is important to underline that the recommended n6/n3 PUFA ratio is estimated to be 4/1, while the dietary n6/n3 PUFA ratio is about 10/1 [32]. In this context, novel dietary sources with low n6/n3 PUFA ratio, among which HSO and food produced from hemp seeds, are in high demand for improving human nutrition, in accordance with European Food Safety Authority (EFSA) recommendations [33]. In this work, the value of this ratio ranged between 3.0 and 4.5 for ITA HSO samples and between 3.0 and 3.6 for EEU samples. These results are in agreement with those reported by other authors [24,26].

The values of AI, TI, and HH are more useful in evaluating the nutritional and health properties of food lipids than the simple FA %composition. They are interesting indicators for heart disease susceptibility, in fact, the lower the value, the lower the risk of disease developing. The values of AI ranged between 0.07 and 0.09 for all samples, while the values of TI were on average 0.12 for all samples. The lowest HH value was found for an Italian sample, while the highest for an EEU sample. Ying et al. (2018) studied the nutritional properties of unconventional cold-pressed edible oils and reported values of 0.09 (AI), 0.23 (TI), 14.88 (HH) for HSO purchased in Poland [21]. Ulbricht and Southgate (1991) showed that the AI of coconut, palm, olive, and sunflower oils were 13.63, 0.88, 0.14, and 0.07 respectively [31]. It can be affirmed that the analyzed HSO samples showed AI values similar to sunflower oil (0.07), and to blackcurrant (0.10) and dill (0.08) seed oils [21]. Significantly higher TI values for coconut (6.18) and palm (2.07) oils, while lower (0.32 and 0.28 for olive and sunflower oils, respectively) were reported by Ulbricht and Southgate (1991) [31]. Ying et al. (2018) reported an HH value of 14.88 for HSO, a value similar to oils obtained from parsley seed (14.21), dill seed (12.56), and blackcurrant seed (13.82) oils, even if the highest value was found for apricot kernel oil (21.49) [21].

Therefore, an HSO enriched diet can be associated with a less atherogenic and thrombogenic diet. This evidence can lead to a decrease in the incidence of coronary heart diseases.

Moreover, UI and COX are useful to evaluate the susceptibility to oxidation due to the presence of UFA. The values of UI ranged between 188.9 and 198.3 for Italian samples, and from 190.6 to 197.2 for EEU samples, while COX values were 8.5–9.5 and 9.5–10.1 for ITA and EEU samples, respectively. Similar COX values were reported by Ratusz et al. (2018) for twenty-nine Camelina oils [30], while higher values (12.03–15.40) were found by Symoniuk et al. (2017) for fifteen Polish cold-pressed linseed oils [34].

In this research, no statistically significant differences in ratios/indexes were found between ITA and EEU samples.

### 3.2. Stereospecific Analysis of TAG of HSO Samples

As a further study, the complete characterization of TAG structure was obtained by an enzymatic procedure, based on the use of DAGK, which allowed the complete TAG stereospecific analysis. It should be taken into consideration that the quali- and quantitative profiles of TAG derive from the enzymatic specificity of the biosynthetic process by providing a fingerprint of origin species. The DAGK method takes into consideration that the FA are specifically esterified in the three *sn*-positions of glycerol structure, as a reflection of the biochemical pathway of TAG. Based on the above, stereospecific analysis data are useful for product characterization, authentication, and geographical discrimination of vegetable and animal foods [14,15,35].

In addition to the interesting total acidic profile, the molecular distribution of single FA on dietary TAG cannot be overlooked for explaining the HSO beneficial properties. During digestion, the FA of the *sn*-1 and *sn*-3 positions of TAG backbone are readily hydrolyzed by pancreatic and lipoprotein lipases [36]. The *sn*-2 position is of great relevance from a nutritional point of view because FA esterified in the *sn*-2 position are highly bioavailable, because *sn*-2-MAG are completely absorbed and re-esterified in the enterocytes. This preservation of the native FA in the *sn-*2 position has an extending effect in other metabolic processes [36].

Table 3 shows the results of the stereospecific analysis for Italian and EEU samples, while Figure S2 (Supplementary Materials) shows the composition of some FA categories (MUFA, PUFA, and EFA) of nutritional importance in TAG *sn*-2 position of ITA and EEU HSO samples.

It can be observed that Italian HSO samples had a high percentage of UFA (99.3–99.6%) in *sn*-2 position, represented by MUFA (16.0–20.6%) and PUFA (78.8–83.5%). Clearly, the presence of EFA (74.4–79.4% for ITA samples; 81.7–85.9% for EEU samples) at the *sn*-2 position of TAG structure gives considerable benefits for long-term health effects. Although there are numerous evidences on the health benefits of PUFA, very few studies have been published, to date, on the nutritional effects of dietary HSO, and even fewer are well-controlled clinical investigations into its health benefits.

The most interesting data was the high value of EFA (sum of LA and ALA) in *sn*-2 position both for ITA samples (74.4–79.4%) and for EEU samples (81.7–85.9%). In this important *sn*-position, the main FA were LA (>64.6% for ITA samples; >67.9% for EEU samples), followed by OA (>15.8% for ITA samples; >12.6% for EEU samples), and ALA (>8.9% for ITA samples; >13.0% for EEU samples). Also in EEU samples UFA are the main fraction in *sn*-2 position, but it is important to observe that GLA is represented in this position in lower percentages (0.6–0.8%) in respect to ITA samples (2.6–3.7%), as well as OA (10.3–16.4% for EEU and 15.8–20.4% for ITA samples). Differently, the SFA % is low (0.43–0.63% for ITA samples, and 0.3–0.4% for EEU samples) in *sn*-2 position (data not shown), while a higher percentage of SFA (21.5–24.3% for ITA samples; 19.2–23.7% for EEU samples) was detected in *sn*-1 position in respect to *sn*-3 position (5.8–11.3% for Italian samples; 7.9–12.2% for Extra-European samples).

For the first time, to the best of our knowledge, the stereospecific analysis data of HSO samples are reported. For this reason the comparison with literature data was carried out considering other LA-rich vegetable oils, among which pumpkin seed (PSO), corn, soybean, sunflower or grapeseed oils [37]. As an example, UFAs are represented in high percentage (98.5%) in *sn*-2 position of PSO samples, but lower than those reported for HSO samples. In PSO samples, MUFA are around 36.2% and PUFA around 62.4%, while in HSO samples PUFA are at least five times higher than MUFA [15]. Also for corn and soybean oils a preference of LA in TAG *sn*-2 position was reported [37].

Regarding HSO, TAG structural data are available, even if they are relative only to *sn*-2 position. Mungure and Birch (2014) carried out the pancreatic lipase treatment, considering the *sn*-1 and *sn*-3 positions equal to each other [23], while Gao and Birch (2016) evaluated the percentage distribution of FA and their positional distribution following Novozym 435 treatment of cold-pressed HSO sample from New Zealand [38]. Mungure and Birch (2014) reported that LA was the most abundant (58.2%) fatty acid at the *sn*-2 position in HSO, followed by OA (18.01%) and ALA (11.52%) [23]. The same authors have also carried out a direct HSO TAG analysis by HPLC and ESI-MS. In this regard, the indirect approach for TAG analysis applied in this study, although time consuming and complex for routine application, shows the advantage to obtain the data of TAG fingerprinting, without the need to use sophisticated and expensive equipment.

In this work, complete stereospecific data showed that SFA are preferentially esterified in *sn*-1 position (21.5–24.3%), in particular PA (14.6–17.0% for ITA samples; 13.5–16.5% for EEU samples), followed by SA (4.9–6.4% for ITA samples; 4.7–6.3% for EEU samples). Regarding TAG *sn*-1 and *sn*-3 positions, OA preferred the *sn*-1 position, while LA and ALA preferred the *sn*-3 position. ALA was more represented in *sn*-3 position (19.0–22.9% for ITA samples; 20.7–27.8% for EEU samples) in respect to *sn*-2 position (8.9–13.2% for ITA samples; 13.0–17.0% for EEU samples). This particular trend was also confirmed by Gao and Birch (2016) [38]. These authors reported that the total % SFA was significantly higher in the *sn*-1(3) positions and the total MUFA and PUFA was significantly high in the *sn-*2 position, as expected. In addition, these authors did not show the presence of SA among SFA in *sn*-2 position, while in all ITA and EEU samples investigated in this work no arachidic, gondoic, and behenic acids were detected in this central position.

From the information reported by Callaway and Pate (2009), it seems that HSO is the only commercial food oil (among flaxseed oil, rapeseed oil, olive oil, corn oil, soybean oil, and sunflower oil) that can deliver both EFA at the *sn*-2 position in significant amounts, as the triacylglycerols ALA-LA-ALA, LA-LA-LA and LA-ALA-ALA make up about 45% of the total found in HSO [39].

Statistically significant differences (*p* ≤ 0.05) were observed between the two groups of samples (ITA and EEU) for PUFA and EFA percentages in *sn*-2 position.

### 3.3. Principal Component Analysis

The chemical composition of foods, as well as organoleptic, nutritional, and bioactive properties, can be influenced by several factors (i.e., geographical origin, variety, cultivation, breeding, and/or feeding conditions). Therefore, the development of accurate and robust analytical methods to guarantee the authenticity and traceability of foods is highly recommended. Multivariate parametric techniques have been used in order to classify and discriminate samples from different origins [14,15,35].

In this paper, principal component analysis was performed on total and intrapositional FA %composition of TAG from ITA and EEU samples. Table 4 shows the obtained eigenvalue, percentage of variance, and cumulative percentage of the principal components. The eigenvalues reflect the quality of the projection from the N-dimensional initial table to a lower number of dimensions.

The first eigenvalue equals 11.6 and represents 50.38% of the total variability, meaning that representing the data on only one axis, the system will be able to see 50.38% of the total variability of the data. The first two components represent 70.68% of the initial variability.

**Figure 1:**
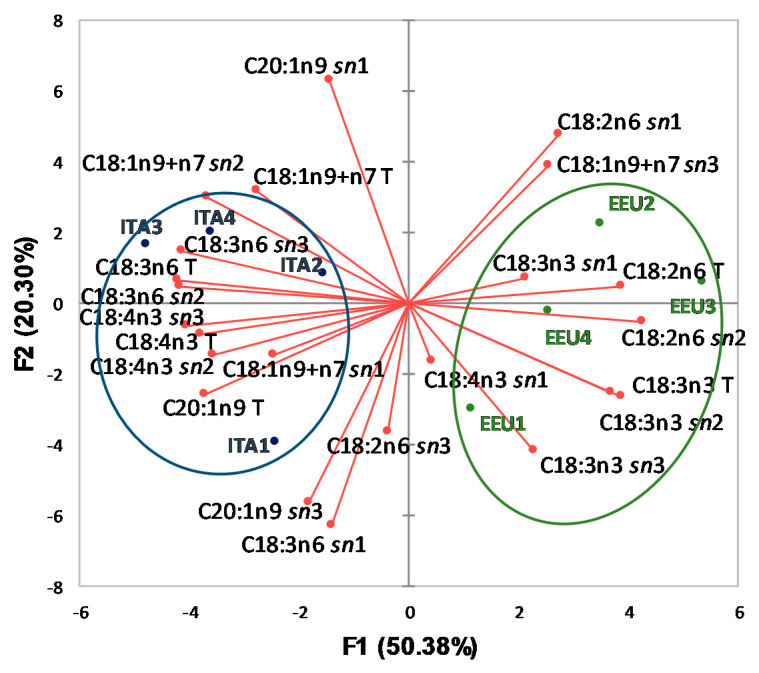
Plot of principal components obtained using total and intrapositional FA % compositions of TAG of HSO samples. ITA, Italian samples; EEU, Extra-European Union samples; C18:1n9+n7, oleic and octadecenoic; C20:1n9, gondoic; C18:2n6, linoleic; C18:3n3, α-linolenic; C18:3n6, γ-linoleic; C18:4n3, stearidonic acids. The letter T after FA indicates that the variable refers to the composition of FA in the total TAG; the abbreviation *sn*1, *sn*2, or *sn*3 after FA indicates that the variable refers to the composition of FA in TAG *sn*-1, *sn*-2 or *sn*-3 position, respectively.

Figure 1 shows the vectors (red) of the investigated variables and the distribution of HSO samples in the plane defined by the first two principal components, according to FA total and intrapositional %compositions. The horizontal axis is the first PCA dimension, representing 50.38% of the initial information, while the vertical axis is the second PCA dimension (20.30% of the initial information).

It can be observed that ITA samples (blue, on the left) were located in a different area of the plane with respect to EEU samples (green, on the right). Axis F1 is linked to some important FA % contents: on the right, essentially two vectors (C18:2n6 T and C18:2n6 *sn*2); on the left, essentially four vectors (C18:3n6 T, C18:4n3 T, and C18:3n6 *sn*2 and C18:4n3 *sn*3). The length of the vectors represents the quality in the investigated PCA dimensions, so, it can be observed that EEU samples were located mainly along the vectors of total FA % content (C18:2n6 T and C18:3n3 T) and along the vectors of intrapositional FA % content (C18:2n6 *sn*1; C18:2n6 *sn*2; C18:3n3 *sn*2; C18:3n3 *sn*3; C18:1n9+n7 *sn*3). For ITA samples, the main vectors useful to the differentiation are represented in particular by: C18:1n9+n7 T, C18:3n6 T, C18:4n3 T, and C20:1n9 T, for total FA % contents; C18:3n6 *sn*1, C20:1n9 *sn*1, C18:1n9+7 *sn*2, C18:3n6 *sn*2, C18:4n3 *sn*2, C18:3n6 *sn*3, for intrapositional FA % content. Some variables are positively correlated (narrow angle of the vectors) as for example C18:3n3 T vs. C18:3n3 *sn*2, and C18:3n6 T vs. C18:3n6 *sn*2, and C18:4n3 T vs. C18:4n3 *sn*3 are positively-linked vectors.

The obtained results showed that total and intrapositional FA % compositions represent interesting fingerprint components of HSO. The applied analytical approach highlights that the results of deep characterization of TAG fraction are possible markers for assessing the authenticity of oil and fat.

## 4. Conclusions

In this work, the complete stereospecific analysis of TAG fraction of HSO samples was carried out for the first time. The results confirm that HSO is an interesting vegetable oil with important nutritional value, related to the presence of MUFA, PUFA, and EFA above all in the *sn*-2 position of lipid structure. The insights gained from this research could help support potential HSO applications with health benefits in the food and nutraceutical sectors. This paper confirms that stereospecific analysis represents a potent analytical investigative tool able to give the fingerprint of TAG fraction, also with the aim of differentiating samples of different geographical origin. Besides these advantages, the data could be useful for the food industry and consumers in order to evaluate HSO quality and to guarantee its authenticity.

## Figures and Tables

**Table 1:** Total % fatty acid composition (minimum, mean, maximum values, % mol, *n* = 4) of HSO samples.

	ITA			EEU			
FA	Min	Mean	Max	Min	Mean	Max	*p*
C16:0	6.5	7.3	8.1	6.5	6.9	7.1	0.33812
C16:1n7	0.1	0.1	0.1	0.1	0.1	0.1	0.39166
C18:0	2.6	2.9	3.0	2.8	2.9	3.1	0.68353
C18:1n9+n7	13.2	15.3	16.6	12.3	14.3	15.1	0.36275
C18:2n6	54.0	54.7	55.4	56.3	56.9	57.1	**0.00141**
C18:3n6	2.4	2.9	3.4	0.6	0.6	0.7	**0.00130**
C20:0	0.6	0.7	0.8	0.4	0.5	0.5	0.11423
C18:3n3	12.3	14.6	17.5	15.8	17.0	19.0	**0.00322**
C20:1n9	0.4	0.4	0.4	0.3	0.3	0.5	**0.00558**
C18:4n3	0.6	0.8	1.0	0.2	0.3	0.3	**0.00787**
C22:0	0.3	0.3	0.4	0.2	0.2	0.2	0.33812

ITA, Italian samples; EEU, Extra-European Union samples; FA, fatty acid; Min, minimum value; Mean, mean value; Max, maximum value; *p*, the level of statistical significance (in bold when it is ≤ 0.01).

**Table 2:** FA ratios and nutritional quality indexes (minimum, mean, maximum values, *n* = 4) of HSO samples.

	ITA			EEU		
FA	Min	Mean	Max	Min	Mean	Max
UFA/SFA	7.6	8.0	8.5	8.2	8.5	8.9
MUFA/SFA	1.3	1.4	1.5	1.2	1.4	1.5
PUFA/SFA	6.1	6.5	7.2	6.8	7.2	7.5
n6/n3 PUFA	3.0	3.8	4.5	3.0	3.4	3.6
AI	0.07	0.10	0.09	0.08	0.10	0.08
TI	0.10	0.10	0.14	0.10	0.10	0.12
HH	10.4	11.8	13.3	12.4	12.9	13.7
UI	188.9	192.0	198.3	190.6	192.5	197.2
COX	8.5	8.9	9.5	9.5	9.7	10.1

SFA: saturated fatty acids; MUFA: monounsaturated fatty acids; PUFA: polyunsaturated fatty acids; UFA: sum of MUFA and PUFA. AI: atherogenic index (C12:0 + 4 × C14:0 + C16:0)/(MUFA + PUFAn6 + PUFAn3); TI: thrombogenic index (C14:0 + C16:0 + C18:0)/(0.5 × MUFA + 0.5 × PUFAn6 + 3 × PUFAn3 + (PUFAn3/PUFAn6)); HH, hypocholesterolemic/hypercholesterolemic index (C18:1 + C18:2 + C18:3 + C18:4 + C20:4)/(C14:0 + C16:0); UI: unsaturation index: ∑(mol % of each FA) × (number of double bonds of each FA); COX, calculated oxidability value (C18:1 + 10.3 × C18:2 + 20.6 × C18:3)/100.

**Table 3:** Intrapositional % fatty acid composition (% mol, mean value ± SD, *n* = 4) of HSO samples.

	**ITA1**			**ITA2**			**ITA3**			**ITA4**		
**FA**	***sn*** **-1**	***sn*** **-2**	***sn*** **-3**	***sn*** **-1**	***sn*** **-2**	***sn*** **-3**	***sn*** **-1**	***sn*** **-2**	***sn*** **-3**	***sn*** **-1**	***sn*** **-2**	***sn*** **-3**
C16:0	16.3±0.1	0.4±0.0	1.0 ± 0.5	15.9 ± 0.1	0.4 ± 0.1	4.9 ± 0.3	17.0 ± 0.0	0.5 ± 0.0	6.1 ± 0.1	14.6 ± 0.0	0.3 ± 0.0	5.5 ± 0.6
C16:1n7	0.1 ± 0.0	0.1 ± 0.0	0.2 ± 0.0	0.1 ± 0.0	0.1 ± 0.0	0.0 ± 0.0	0.1 ± 0.0	0.1 ± 0.0	0.0 ± 0.1	0.1 ± 0.0	0.1 ± 0.0	0.1 ± 0.0
C18:0	6.4 ± 0.0	0.1 ± 0.0	2.5 ± 0.1	5.7 ± 0.0	0.1 ± 0.0	2.8 ± 0.0	4.9 ± 0.0	0.1 ± 0.0	2.6 ± 0.0	6.0 ± 0.0	0.1 ± 0.0	2.8 ± 0.0
C18:1n9+n7	17.4 ± 0.1	15.8 ± 0.3	6.4 ± 1.1	20.8 ± 0.1	18.6 ± 0.7	6.8 ± 2.2	22.3 ± 0.0	20.4 ± 0.7	5.8 ± 0.8	20.7 ± 0.1	20.4 ± 0.5	8.8 ± 1.0
C18:2n6	40.9 ± 0.2	66.1 ± 0.4	56.8 ± 1.5	44.7 ± 0.2	66.9 ± 0.6	54.4 ± 2.0	43.9 ± 0.1	65.5 ± 0.4	54.4 ± 0.3	45.8 ± 0.0	64.6 ± 0.4	49.5 ± 0.2
C18:3n6	0.4 ± 0.0	3.2 ± 0.0	4.7 ± 0.0	0.1 ± 0.0	2.6 ± 0.0	4.4 ± 0.0	0.1 ± 0.0	3.7 ± 0.1	6.8 ± 0.0	-	3.0 ± 0.0	5.7 ± 0.0
C20:0	0.4 ± 0.0	-	2.2 ± 0.1	0.0 ± 0.0	-	2.0 ± 0.0	-	-	2.1 ± 0.0	-	-	2.2 ± 0.0
C18:3n3	15.8 ± 0.0	13.2 ± 0.1	22.9 ± 0.4	11.9 ± 0.0	10.7 ± 0.1	21.9 ± 0.6	10.7 ± 0.1	8.9 ± 0.1	19.0 ± 0.4	11.5 ± 0.1	10.8 ± 0.1	22.5 ± 0.2
C20:1n9	-	-	1.4 ± 0.0	0.3 ± 0.0	-	0.8 ± 0.0	0.5 ± 0.0	-	0.9 ± 0.1	0.4 ± 0.0	-	1.0 ± 0.0
C18:4n3	0.1 ± 0.0	1.0 ± 0.0	1.8 ± 0.1	-	0.6 ± 0.0	1.4 ± 0.0	-	0.7 ± 0.0	1.7 ± 0.0	-	0.7 ± 0.0	1.7 ± 0.0
C22:0	1.2 ± 0.0	-	-	0.4 ± 0.0	-	0.6 ± 0.0	0.6 ± 0.0	-	0.5 ± 0.0	0.9 ± 0.0	-	0.4 ± 0.0
	**EEU1**			**EEU2**			**EEU3**			**EEU4**		
**FA**	***sn*** **-1**	***sn*** **-2**	***sn*** **-3**	***sn*** **-1**	***sn*** **-2**	***sn*** **-3**	***sn*** **-1**	***sn*** **-2**	***sn*** **-3**	***sn*** **-1**	***sn*** **-2**	***sn*** **-3**
C16:0	16.5 ± 0.2	0.3 ± 0.0	5.2 ± 0.2	13.5 ± 0.1	0.3 ± 0.0	7.1 ± 0.3	14.1 ± 0.1	0.3 ± 0.0	6.0 ± 0.1	15.2 ± 0.1	0.3 ± 0.0	3.7 ± 0.0
C16:1n7	0.1 ± 0.0	0.1 ± 0.0	0.1 ± 0.1	0.2 ± 0.0	0.1 ± 0.0	-	0.2 ± 0.0	0.1 ± 0.0	-	0.1 ± 0.0	0.1 ± 0.0	0.1 ± 0.0
C18:0	6.3 ± 0.0	0.1 ± 0.0	3.2 ± 0.2	5.0 ± 0.0	0.1 ± 0.0	3.8 ± 0.0	4.7 ± 0.1	0.1 ± 0.0	3.2 ± 0.0	5.7 ± 0.0	0.1 ± 0.0	3.1 ± 0.0
C18:1n9 + n7	23.9 ± 0.2	16.4 ± 0.0	5.3 ± 0.6	16.6 ± 0.1	17.0 ± 0.2	12.1 ± 0.0	13.8 ± 0.1	12.6 ± 0.1	11.1 ± 0.1	20.7 ± 0.0	15.9 ± 0.0	6.4 ± 0.0
C18:2n6	44.3 ± 0.0	67.9 ± 0.1	53.2 ± 1.6	48.0 ± 0.0	68.7 ± 0.2	52.8 ± 0.3	48.5 ± 0.1	69.0 ± 0.0	51.1 ± 0.2	43.8 ± 0.1	68.5 ± 0.0	57.0 ± 1.6
C18:3n6	0.3 ± 0.1	0.6 ± 0.0	0.7 ± 0.1	0.0 ± 0.0	0.7 ± 0.0	1.1 ± 0.0	-	0.7 ± 0.0	1.2 ± 0.0	-	0.8 ± 0.1	1.3 ± 0.0
C20:0	-	-	2.0 ± 0.0	-	-	1.3 ± 0.0	-	-	0.9 ± 0.0	-	-	0.9 ± 0.0
C18:3n3	7.8 ± 0.1	14.3 ± 0.0	27.8 ± 0.3	15.3 ± 0.1	13.0 ± 0.1	20.7 ± 0.2	18.1 ± 0.0	17.0 ± 0.0	24.7 ± 0.0	12.5 ± 0.2	14.0 ± 0.1	25.8 ± 0.0
C20:1n9	-	-	1.4 ± 0.0	0.3 ± 0.0	-	0.7 ± 0.0	0.2 ± 0.0	-	0.7 ± 0.0	0.3 ± 0.0	-	0.7 ± 0.0
C18:4n3	-	0.2 ± 0.0	0.7 ± 0.0	0.1 ± 0.0	0.2 ± 0.0	0.4 ± 0.0	-	0.3 ± 0.0	0.7 ± 0.0	-	0.3 ± 0.0	0.7 ± 0.0
C22:0	0.9 ± 0.0	-	0.5 ± 0.1	0.9 ± 0.0	-	-	0.4 ± 0.0	-	0.3 ± 0.0	0.6 ± 0.0	-	0.2 ± 0.0

-: <0.1%.

**Table 4:** PCA: eigenvalue, percentage of variance, and cumulative percentage of principal components (F1–F7).

	F1	F2	F3	F4	F5	F6	F7
Eigenvalue	11.587	4.669	3.521	1.712	1.157	0.290	0.064
Variability (%)	50.378	20.298	15.311	7.445	5.030	1.259	0.279
Cumulative (%)	50.378	70.676	85.987	93.431	98.462	99.721	100.000

## Data Availability

Data contained within the article or Supplementary Materials are here presented for the first time.

## Supplementary Materials

The following are available online at https://www.mdpi.com/article/10.3390/foods10050916/s1, Figure S1: composition (% mol) of FA categories (SFA, UFA, MUFA, PUFA, n3 PUFA, n6 PUFA) of the HSO samples. ITA1-4, Italian samples; EEU, Extra-European Union samples. SFA, saturated; UFA, unsaturated; MUFA, monounsaturated; PUFA, polyunsaturated fatty acids, Figure S2: composition (% mol) of FA categories in TAG *sn*-2 position of HSO samples: ITA1-4, Italian samples; EEU1-4, Extra-European Union samples. MUFA, monounsaturated fatty acids; PUFA, polyunsaturated fatty acids; EFA, essential fatty acids.
