# Supplementary material for: Characterization of the Triacylglycerol Fraction of Italian and Extra-European Hemp Seed Oil
Source: Foods. 2021 Apr 22;10(5):916. doi: 10.3390/foods10050916 (PMC8143575; doi:10.3390/foods10050916)
Supplement: Supplementary file 1 [file foods-10-00916-s001.zip › foods-1180046-supplementary.pdf]

*Article*

# Characterization of Triacylglycerol Fraction of Italian and Extra-European Hemp Seed Oil

**Carmela Tringaniello [1], Lina Cossignani [1,2]* and Francesca Blasi [1]**

[1] University of Perugia, Department of Pharmaceutical Sciences, 06100 Perugia (Italy); carmela.tringaniello@unipg.it (C.T.); francesca.blasi@unipg.it (F.B.); lina.cossignani@unipg.it (L.C.)
[2] Center for Perinatal and Reproductive Medicine, University of Perugia, Santa Maria della Misericordia Uni-versity Hospital, Sant'Andrea delle Fratte, 06132 Perugia, Italy
* Correspondence: lina.cossignani@unipg.it (L.C.); Tel.: +39-755857959

**SUPPLEMENTARY MATERIAL**

**FIGURE 1S**

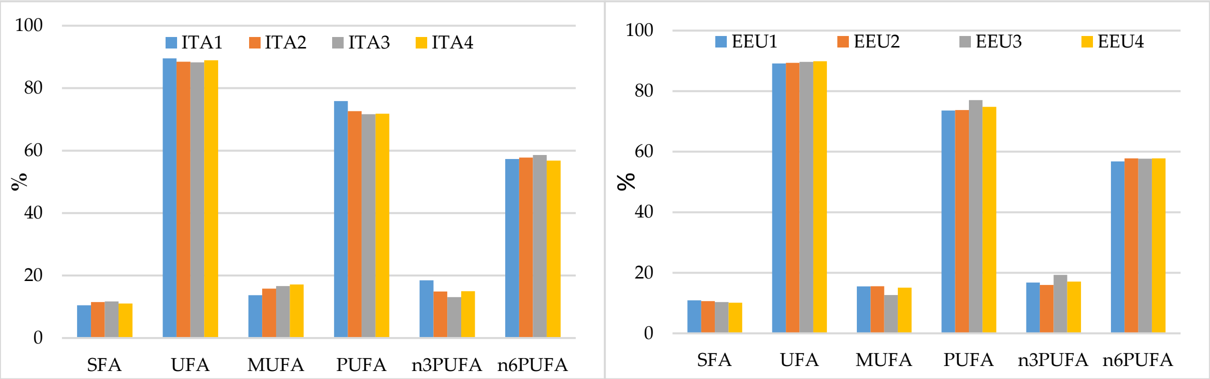


**Figure 1S.** Composition (% mol) of FA categories (SFA, UFA, MUFA, PUFA, n3PUFA, n6PUFA) of the HSO samples. ITA1-4, Italian samples; EEU, Extra-European Union samples. SFA, saturated, UFA, unsaturated, MUFA, monounsaturated, PUFA, polyunsaturated fatty acids

**FIGURE 2S**

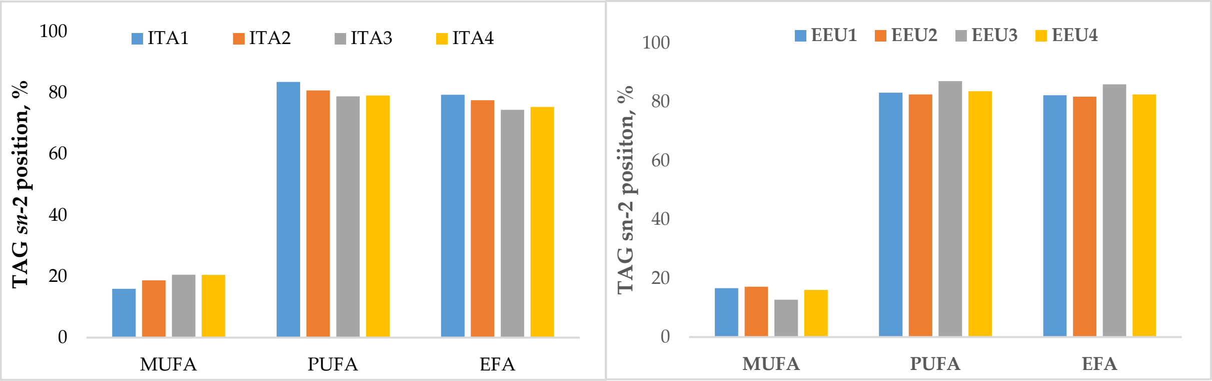


**Figure 2S.** Composition (% mol) of FA categories in TAG sn-2 position of HSO samples: ITA1-4, Italian samples; EEU1-4, Extra-European Union samples. MUFA, monounsaturated fatty acids; PUFA, polyunsaturated fatty acids; EFA, essential fatty acids
